# Supplementary material for: Overcrowded housing reduces COVID-19 mitigation measures and lowers emotional health among San Diego refugees from September to November of 2020
Source: PLoS One. 2023 Jun 20;18(6):e0286993. doi: 10.1371/journal.pone.0286993 (PMC10281572; doi:10.1371/journal.pone.0286993)
Supplement: Attachment — Submitted filename: Review_PLOSONE_8.22.22.docx [file pone.0286993.s001.docx]

PLOS ONE

Review

August 22, 2022

Title: Overcrowded Housing Reduces COVID-19 Mitigation Measures and Increases Poor

Emotional Health Among San Diego Refugees

This manuscript is on an important topic for refugee health. The analysis is meaningful and would be an important contribution to the literature. However, the manuscript needs substantial revisions throughout. Below please find some feedback.

- Title
  - I suggest using more of an objective word like low instead of the subjective word poor in the title and throughout.
  - I suggest adding the time period in the title as outcomes change throughout time.
- Abstract
  - The total sample size is 544, but it reads as half the sample size in the abstract. I suggest re-writing this to make it clearer.
  - I suggest replacing “fewer” odds with lower odds.
- Introduction
  - I suggest adding bolded subheadings throughout the introduction to guide the reader.
  - A reference is needed for the first sentence.
  - There are a couple of run-on sentences at the end of the first paragraph.
  - Some references are numbered and some are authors last name.
  - Period missing at the end of the introduction section.
- Methods
  - The STROBE guidelines should be used to make the methods section clear and consistent with previous literature. Sections should include setting, participants, variables, data source, and statistical methods. <https://www.equator-network.org/wp-content/uploads/2015/10/STROBE_checklist_v4_combined.pdf>
  - “Interviews were limited to no more than two individuals per household (typically a parent and an adult child).” – How many interviews were completed with individuals from the same household? This should be mentioned as a limitation in the discussion.
  - How many questions were on the survey? About how long did it take for participants to complete?
  - The statistical methods are mentioned before describing the survey and variables, which should come first.
  - “Data gathered from the questionnaires was anonymous” – How do you know if a participants completed the survey more than once? This could be mentioned in the limitations.
  - Data is based on self-report, which should be described in the limitations.
  - What options were given for gender? Were there responses available for: prefer not to respond, non-binary, or transgender?
- Results
  - The first paragraph of the results jumps into a comparison without first describing the overall population of respondents. How old were the respondents for example? What regions or countries are respondents from? What percent of respondents are refugees versus asylum seekers? How many of the respondents are refugees/asylees versus children of refugees/asylees born in the US?
  - An overall demographics table would be valuable as an initial table.
  - This sentence describes methods and should be moved to the methods section: “In consultation with PANA leadership, the study team decided to dichotomize this variable and compare participants who reported ‘never’ experiencing emotional problems vs all others.”
  - This sentence interprets the results and should be moved to the discussion section: “Discussing mental health is highly stigmatized in the community, and the study team agreed that disclosing any concerns related to emotional or mental health in this context was likely indicative of significant levels of emotional distress.”
- Discussion
  - I suggest adding bolded subheadings throughout the discussion to guide the reader.
  - I suggest re-wording the findings to describe how more support is needed for this population as opposed to it reading as if this population is not taking care of their health and putting others at risk.
  - The second paragraph of the discussion should be moved to a limitations section at the end of the discussion before the conclusions.
  - More of a description of the time period is needed related to COVID-19 and testing. For example, how accessible were COVID-19 testing at that time? When did the government send out home tests for free?
  - This sentence implies causality instead of an association: “housing conditions can add an extra emotional and mental burden”
  - United States and US are used. I suggest writing out in full each time.
  - “San Diegan” – Perhaps re-word to refugees living in San Diego.
  - A limitations section was missing.
  - It would be great to see more specifics about next steps related to affordable housing mentioned in the conclusions. What are some specific steps that could be taken to address this issue? Have other counties, states, or countries successfully found affordable housing solutions?
- Tables and Figures
  - Tables should include more comprehensive titles that answer who (refugee and asylum seekers), what, where (San Diego County, CA), and when (time period).
  - I suggest consistency in the number of significant digits in the p values in the tables.
