# Supplementary material for: Overcrowded housing reduces COVID-19 mitigation measures and lowers emotional health among San Diego refugees from September to November of 2020
Source: PLoS One. 2023 Jun 20;18(6):e0286993. doi: 10.1371/journal.pone.0286993 (PMC10281572; doi:10.1371/journal.pone.0286993)
Supplement: Attachment — Submitted filename: Response to Reviewers.docx [file pone.0286993.s002.docx]

Herbert Wertheim School of Public Health

University of California, San Diego

9500 Gilman Dr. CRSF #27

La Jolla, CA 92093

Letter to the Editors:

Thank you all for your feedback and suggestions.

## Reviewer #1:

This is a carefully written, thoughtfully designed study. It adds nuance to our understanding of SDoH for newcomers. It contributes to a body of literature on mitigating future pandemics.

### Major concern:

1. I believe the survey protocol allowed up to 2 respondents per household. Does this mean the ~500 respondents might represent ~250 households? If so, would this not require attention in the model, e.g., household as a fixed effect?

Response: This is an important observation, and we agree with the reviewer’s critique. While up to 2 individuals were technically eligible per household, it was rare that more than one person actually participated in reality. In large part because it was rare, household-level data were not collected and so we are not able to include a mixed effect in the model. We have acknowledged this flaw in our data with the following text added to the limitations section:

*Furthermore, although the survey was limited to two participants per household, the survey did not record how many participants belonged in the same household. Therefore, we were unable to adjust for a household size as a fixed effect. However, PANA interviewers reported that in practice it was extremely for two respondents from the same household to participate in the survey.*

### Minor questions:

2. Could the authors clarify the use of the term "refugee" to characterize survey respondents? Based on the description of PANA and community venue-based recruitment, it sounds like respondents might be refugees, asylum-seekers, or others who speak the same languages but have arrived in the US via family sponsorship, work, etc.

*Response: All families interviewed are either recent arrivals or folks who have become new Americans and all arrived as refugees. Youth interviewed might have been born in the U.S. but their parents arrived as refugees. We have clarified by adding the text below:*

*“Refugee community member” was construed broadly and included individuals who had arrived as refugees or asylum seekers or their American born children”*

3. Could the authors add a comment regarding the low employment rate?

Response: Thank you for this observation – we have expanded the discussion to include a brief discussion of what we agree is a surprisingly low employment rate. The following text has been added:

*Just over 1 in 4 individuals reported engaging in work for money in the previous 2 weeks. While some of this number may be an artifact of sampling bias (i.e., those who responded were more likely to be free during the day to participate in the survey) and/or represent individuals who are full time students or homemakers, previous work conducted by our team demonstrated that the refugee community experienced extremely high rates of job loss during the pandemic*

4. I appreciate the description of weekly meetings to address translation challenges. Could the authors offer a more detailed description, e.g., as an appendix? It would be helpful to know more about the language and cultural qualifications (formal or informal) of the Arabic and Spanish translators and the steps study team members might have taken to ensure equivalence, e.g., meetings to review the English versions, proof readers, etc. Additionally, it sounds as if many languages were sight-translated from an English version of the survey? How were those team members trained to ensure comprehension of the meaning and intention of the English survey items? How did they communicate mental health vocab or other challenging vocab where linguistic and community equivalent terms may be harder to find? Did they develop a glossary for key terms?

*Response: PANA staff initially translated the survey into Somali and Arabic through outside consultants. PANA staff engaged in two or three training sessions discussing the survey questions with the primary researchers to understand what words mean. Because staff come from the communities we serve and speak the language, they were able to conduct the interviews and help folks understand what we mean.*

5. Is there a reason the authors elected not to share data on differences between language groups or include language in the adjusted model? I realize this is a nuanced decision. But communities who speak languages of lesser diffusion may face enhanced barriers to Section 8 applications.

*Response: We agree with this observation. The decision was a practical one driven primarily by statistical necessity. We conducted qualitative work concurrent with the community survey which allowed us to explore these topics with more nuance (see:* *https://www.panasd.org/refugee-experiences-report). Because language and region of origin maps on to historic waves of refugee resettlement in San Diego, which in turn correlates with specific areas of the county in which individuals settled, primary language group is also significantly confounded by year of arrival and acculturation. For the purposes of this manuscript, we therefore chose to focus on experiences that were common across the refugee community, in concert with PANA’s mission to build community power through solidarity.*

## Reviewer #2:

This manuscript is on an important topic for refugee health. The analysis is meaningful and would be an important contribution to the literature. However, the manuscript needs substantial revisions throughout. Below please find some feedback.

*Response: Thank you for your feedback. Your suggestions have been noted and have strengthened this manuscript.*

### Title

o I suggest using more of an objective word like low instead of the subjective word poor in the title and throughout.

*Response: We have replaced the word “poor” with the word “low.” Thank you for the suggestion.*

o I suggest adding the time period in the title as outcomes change throughout time.

*Response: We have added “from September to November of 2020” to the title.*

### Abstract

o The total sample size is 544, but it reads as half the sample size in the abstract. I suggest re-writing this to make it clearer.

*Response: We have re-wrote the abstract to match the correct sample size.*

o I suggest replacing “fewer” odds with lower odds.

*Response: We have replaced “fewer” with “lower” odds.*

### Introduction

o I suggest adding bolded subheadings throughout the introduction to guide the reader.

*Response: We have added bolded subheadings throughout the introduction.*

o A reference is needed for the first sentence.

*Response: We have added a reference to the first sentence.*

o There are a couple of run-on sentences at the end of the first paragraph.

*Response: We have reformatted the sentences so that they flow better and are no longer run-on sentences.*

o Some references are numbered and some are authors last name.

*Response: We have properly reformatted the references throughout the manuscript.*

o Period missing at the end of the introduction section.

*Response: We have added the missing period. Thank you for catching that error!*

### Methods

o The STROBE guidelines should be used to make the methods section clear and consistent with previous literature. Sections should include setting, participants, variables, data source, and statistical methods. <https://www.equator-network.org/wp-content/uploads/2015/10/STROBE_checklist_v4_combined.pdf>

*Response: We have incorporated relevant STROBE guidelines to restructure the Methods section.*

o “Interviews were limited to no more than two individuals per household (typically a parent and an adult child).” – How many interviews were completed with individuals from the same household? This should be mentioned as a limitation in the discussion.

*Response: Our partners who conducted the interviews kept track of who was interviewed. However, we did not record how many households had more than one participant in the study. We have added this to the limitations section.*

o How many questions were on the survey? About how long did it take for participants to complete?

*Response: The survey contained 83 questions regarding demographics, COVID-19, housing, employment, health, children, belonging, and resilience. The survey took approximately 30 minutes to complete and up to 1 hour if it was being translated.*

o The statistical methods are mentioned before describing the survey and variables, which should come first.

*Response: We have moved the survey/variable descriptions before the statistical methods section.*

o “Data gathered from the questionnaires was anonymous” – How do you know if a participants completed the survey more than once? This could be mentioned in the limitations.

*Response: “PANA staff worked from a list of families. Each staff interviewer was assigned a list of people based on language. Staff interviewed and conducted the survey by phone with each individual ensuring no duplication.” We added a couple sentences to clarify in the methods section under participants.*

o Data is based on self-report, which should be described in the limitations.

*Response: We have added this to the limitations section.*

o What options were given for gender? Were there responses available for: prefer not to respond, non-binary, or transgender?

*Response: There were only options given for male and female to preserve statistical significance.*

### Results

o The first paragraph of the results jumps into a comparison without first describing the overall population of respondents. How old were the respondents for example? What regions or countries are respondents from? What percent of respondents are refugees versus asylum seekers? How many of the respondents are refugees/asylees versus children of refugees/asylees born in the US?

*Response: We have addressed each of these questions in Table 1. However, we did not record which respondents were refugees/asylees versus children of refugees/asylees born in the US.*

o An overall demographics table would be valuable as an initial table.

*Response: We have created a demographics table (Table 1).*

o This sentence describes methods and should be moved to the methods section: “In consultation with PANA leadership, the study team decided to dichotomize this variable and compare participants who reported ‘never’ experiencing emotional problems vs all others.”

*Response: We have moved this sentence to the location you suggested.*

o This sentence interprets the results and should be moved to the discussion section: “Discussing mental health is highly stigmatized in the community, and the study team agreed that disclosing any concerns related to emotional or mental health in this context was likely indicative of significant levels of emotional distress.”

*Response: We have removed this interpretive sentence from the results section.*

### Discussion

- I suggest adding bolded subheadings throughout the discussion to guide the reader.
  - *Response: We have added bolded subheadings throughout the discussion.*
- I suggest re-wording the findings to describe how more support is needed for this population as opposed to it reading as if this population is not taking care of their health and putting others at risk.
  - *Response: Thank you for this suggestion. We have reworded the findings so as to not suggest the risks faced by this group are because of their choices but due to structural issues.*
- The second paragraph of the discussion should be moved to a limitations section at the end of the discussion before the conclusions.
  - *Response: We have revised the discussion so that this section has been moved to the limitations section prior to the conclusion.*

o More of a description of the time period is needed related to COVID-19 and testing. For example, how accessible were COVID-19 testing at that time? When did the government send out home tests for free?

*Response: This study was conducted during the Fall of 2020. Testing was widely available at clinics and testing centers but antigen “home tests” were not yet available in meaningful amounts. It was not until the first year of the Biden Administration that the government had the capacity to send home tests to households.*

o This sentence implies causality instead of an association: “housing conditions can add an extra emotional and mental burden”

*Response: We have modified this sentence so that it does not imply causality.*

o United States and US are used. I suggest writing out in full each time.

*Response: We have replaced “US” with “United States”.*

o “San Diegan” – Perhaps re-word to refugees living in San Diego.

*Response: We have replaced “San Diegan refugees” with “refugees living in San Diego.”*

o A limitations section was missing.

*Response: We have added a limitation section and have added subheaders.*

o It would be great to see more specifics about next steps related to affordable housing mentioned in the conclusions. What are some specific steps that could be taken to address this issue? Have other counties, states, or countries successfully found affordable housing solutions?

*Response:Thank you for your comment, we have added the following statement in our conclusion:*

*In addition, we recommend the federal government increase the number of Section 8 vouchers available so that wait times are reduced (34). Finally, we recommend that the State of California create legislation and provide funding for permanent social housing that refugee populations can access (10).*

*These policy recommendations and more can be found in the PANA report.*

### Tables and Figures

1. Tables should include more comprehensive titles that answer who (refugee and asylum seekers), what, where (San Diego County, CA), and when (time period).

*Response: We have included comprehensive titles to our tables as you have suggested.*

2. I suggest consistency in the number of significant digits in the p values in the tables.

*Response: We have standardized the number of significant digits in the table p-values.*

## Reviewer #3:

- A very nice community-engaged applied research study addressing an extremely important topic.

*Response: Thank you for this comment.*

### Suggestions:

1. I suggest that they re-arrange Table 1 so that they report measures in the table in the same order in which they report them in the text.

*Response: We added a new demographics table (Now Table 1) and have rearranged the table you were originally referencing (Now Table 2) to report measures in the same order in which we report them in the text*

Best,

Co-author [Corresponding]

Ashkan Hassani, BS

858-842-8315

ahassani@health.ucsd.edu

Lead Field Researcher, Herbert Wertheim School of Public Health

University of California, San Diego

Co-author

Vinton Omaleki, MPH

Qualitative Researcher, Herbert Wertheim School of Public Health

University of California, San Diego
