# Supplementary material for: Overcrowded housing reduces COVID-19 mitigation measures and lowers emotional health among San Diego refugees from September to November of 2020
Source: PLoS One. 2023 Jun 20;18(6):e0286993. doi: 10.1371/journal.pone.0286993 (PMC10281572; doi:10.1371/journal.pone.0286993)
Supplement: Attachment — Submitted filename: Review_PLOSONE_11.30.22.docx [file pone.0286993.s003.docx]

PLOS ONE - Review

November 30, 2022

Title: Overcrowded Housing Reduces COVID-19 Mitigation Measures and Lowers

Emotional Health Among San Diego Refugees from September to November of 2020

This manuscript is on an important topic for refugee health. The analysis is meaningful and would be an important contribution to the literature. Below please find some feedback.

- Abstract
  - I suggest deleting the word “extremely.”
  - Grammatical errors are included throughout the abstract including missing preposition and space.
  - “Odds” should be used not “likely” when using odds ratios.
- Introduction
  - I suggest adding descriptive bolded subheadings throughout the introduction to guide the reader besides background.
  - I suggest a more specific objectives section that answers who, what, where, and when.
- Methods
  - The study was a cross-sectional study design, which was missing from the study design section.
  - This seems to fit better under the participant section: “Individuals were eligible

to participate in the study if they were over the age of 18, able to participate in a language spoken by a trained research assistant, willing to provide informed consent, and part of the refugee community. “Refugee community member” was construed broadly and included individuals who had arrived as refugees or asylum seekers or their American born children.”

- Results
  - Table 1 figure title was missing who (the population), where (CA), and when (timeframe). I suggest writing out Southeast and East.
  - “Odds” should be used not “likely” when using odds ratios.
  - Table 2 figure title – I suggest including refugee participants to the title. I suggest using the same number of significant digits in the p values. I usually see n (%) not % (n) in tables, which you may considering changing.
  - Table 3 – Perhaps add an asterisk for the odds ratios that are statistically significant.
- Discussion
  - I suggest adding more descriptive bolded subheadings throughout the discussion to guide the reader.
  - “Odds” should be used throughout the discussion not “likely” when using odds ratios as the methods.
  - “There is a strong effect size” – This language does not match the statistical methodology
  - I suggest deleting “extremely” in extremely high rates of job loss.
  - I suggest adding only two gender responses as a limitation.
  - The conclusions section is fairly repetitive of the discussion. I suggest adding more specific future directions in this section.
