# Supplementary material for: Overcrowded housing reduces COVID-19 mitigation measures and lowers emotional health among San Diego refugees from September to November of 2020
Source: PLoS One. 2023 Jun 20;18(6):e0286993. doi: 10.1371/journal.pone.0286993 (PMC10281572; doi:10.1371/journal.pone.0286993)
Supplement: Attachment — Submitted filename: Response to Reviewers.docx [file pone.0286993.s004.docx]

Herbert Wertheim School of Public Health

University of California, San Diego

9500 Gilman Dr. CRSF #27

La Jolla, CA 92093

Thank you all for your feedback and suggestions.

### Reviewer #2

Abstract

o I suggest deleting the word “extremely.”

Response: We have removed the word “extremely” from the abstract.

o Grammatical errors are included throughout the abstract including missing prepositions and space.

Response: We have changed all identifiable missing spaces and prepositions.

o “Odds” should be used not “likely” when using odds ratios.

Response: We have removed the word “likely” when discussing odds ratios in the Abstract. Please see highlighted corrections in the manuscript.

Introduction

o I suggest adding descriptive bolded subheadings throughout the introduction to guide the reader besides the background.

Response: We defer to the editor for the preferred format of the manuscript.

o I suggest a more specific objectives section that answers who, what, where, and when.

Response: We believe that delving into too much detail would be redundant in this section.

Methods

o The study was a cross-sectional study design, which was missing from the study design section.

Response: We now have mentioned that this is a cross-sectional study in the study design section.

o This seems to fit better under the participant section: “Individuals were eligible

to participate in the study if they were over the age of 18, able to participate in a language spoken by a trained research assistant, willing to provide informed consent, and part of the refugee community. “Refugee community member” was construed broadly and included individuals who had arrived as refugees or asylum seekers or their American-born children.”

Response: We have moved the following section to the “participants” section. To reduce redundancy we also removed the following sentence from the participant section: “To be eligible to participate in the survey individuals had to be 18 years old or above and identify as a refugee, asylum seeker, or the child of a refugee or asylum seeker.”

Results

o Table 1 figure title was missing who (the population), where (CA), and when (timeframe). I suggest writing out Southeast and East.

Response: We have adjusted the table title to: “Sample Demographics of the refugee participants in San Diego, CA, sampled between November 2020-December 2020. Includes age, average years in the United States, English spoken at home, and gender stratified by region of origin.”

o “Odds” should be used not “likely” when using odds ratios.

Response: We have removed the word “likely” when discussing odds ratios in the manuscript. Please see highlighted corrections in the manuscript.

o Table 2 figure title – I suggest including refugee participants to the title. I suggest using the same number of significant digits in the p values. I usually see n (%) not % (n) in tables, which you may consider changing.

Response: We have adjusted the title. We have also switched the “n” and “%” in the table. The p values sig figs have also been addressed

o Table 3 – Perhaps add an asterisk for the odds ratios that are statistically significant.

Response: We have added asterisks where there is statistical significance. Thank you for your suggestion.

Discussion

o I suggest adding more descriptive bolded subheadings throughout the discussion to guide the reader.

Response: We defer to the editor for the preferred format of the manuscript.

o “Odds” should be used throughout the discussion not “likely” when using odds ratios as the methods.

Response: We have removed the word “likely” when discussing odds ratios in the discussion. Please see highlighted corrections in the manuscript.

o “There is a strong effect size” – This language does not match the statistical methodology

Response: We have replaced the word “strong effect size” with “significant association” to reflect our statistical methodology.

o I suggest deleting “extremely” in extremely high rates of job loss.

Response: We have now deleted all mentions of the word “extremely.”

o I suggest adding only two gender responses as a limitation.

Response: We have added the following statement in the limitations section: “Additionally, gender was limited to two options (male or female) to preserve statistical significance and we are aware that this may not be a true representation of the diverse population.”

o The conclusions section is fairly repetitive in the discussion. I suggest adding more specific future directions in this section.

Response: We have added a specific future direction for research: “Future directions in research should look at the impact of master leasing, housing vouchers, and universal basic income on refugee housing security and general health outcomes.”

### Reviewer #4

Reviewer #4: Thank the researchers for making the necessary corrections to the suggestions of the referees.

We have a few more suggestions for the development of this well-written study that will benefit the literature.

1. Some abbreviations are not mentioned in the article (HUD, E, SE)

Response: Thank you for catching that. We have removed these abbreviations and fully written them out.

2. The type of study that the research belongs to should be stated in the method section.

Response: We have defined this study as a cross-sectional study and have now added that term to the study design section of the methods.

3. Although the researchers state that they follow the STROBE guideline, the article does not contain how the sample size is calculated. In addition, since researchers make use of statistical analysis methods, they should specify the statistical analysis methods they use in the method section by creating a separate section.

Response: The following description of sample size considerations is now included in the methods section:

Data were collected as part of a programmatic report intended to provide a general overview of the community. As such, we did not conduct *a priori* power calculations focused on one specific outcome.

Per recommendations from the STROBE guidelines, we have elected to use confidence intervals to contextualize the precision of our results, rather than conducting post-hoc power calculations(1,2).

4. In Table 1, n's should be written in small letters. N is the frequency in the population, and n is the frequency in the sample.

Response: Thank you, Table 1 has been edited to reflect the suggested changes.

5. While the researchers stated that they only used simple logistic regression analysis, Table 2 contains different statistical analyzes (Chi-square, Mann Whitney U?). The tests used should be indicated by marking the relevant p values in the method section and under the Table. The normality tests or methods of continuous data and descriptive statistics should also be specified in the method.

Response: We appreciate this point, we have added the following text to the methods for clarity:

“We first examined basic univariate frequencies as a study team to develop a basic understanding of sample demographics and the prevalence of indicators of interest within the sample. We then used chi-square and student’s t-tests to test the hypothesis that our primary predictor and covariates of interest were significantly associated with living in severely overcrowded housing.”

6. In Table 1, continuous data are indicated as median (range), while in Table 2 they are indicated as mean (SD). Researchers should indicate how they changed the descriptives.

Response: Thank you. Table 1 has been amended to be more consistent with Table 2.

7. Age and Years in the United States are considered to be continuous data in Table 1 but are shown as % (n). This confusion must be cleared.

Response: Thank you for the input, we have edited the table to be less confusing.

8. In Table 3, under the table, which variables are added to the multivariate model should be stated and the R2 value should be added.

Response: We have added more detail regarding model fit statistics:

“The fully adjusted model is shown in Table 3. The model had a relatively good fit, with a pseudo-r-squared value of 0.20, no specification errors identified using the *linktest* command in Stata (_hatsq p = 0.68), a non-significant Hosmer-Lemeshow goodness-of-fit test (p=0.78).”

9. If the multivariable logistic regression model is used, the method by which it is used (Backward, Enter?), and the tests that examine the suitability of the model should also be added to the method.

Response: Variables were chosen based on theoretical considerations, per Aneshensel’s recommendations (3), and in dialogue with the leadership of the community organization which led the data collection efforts. Our team decided that utilizing step-wise regressions for model design would be inappropriate for a model utilizing a hypothesis and theory-driven approach.

10. The researchers stated that there were 83 questions in the questionnaire, but not all variables were included in the regression analysis. The criteria for inclusion in the model should be specified.

Response: Variables were chosen based on the specific associations of interest (crowded housing and mental health, crowded housing, and testing behaviors). Additional covariates were added to address potential issues of confounding. We have clarified this by adding the following language to the methods section:

“Variables for the present analyses were chosen in consultation with PANA leadership to address the primary research question: What is the association between housing and COVID-19 vulnerability? Vulnerability was broadly understood as both healthcare access and the broader mental health impact of the epidemic. The full report with all variables can be found elsewhere.”

11. The reference categories of categorical variables should be indicated in Table 3.

Response: As the majority of variables in Table 3 are binary we elected not to include the reference variable in order to be parsimonious (i.e., “Never accessed covid test” vs “Ever accessed covid test”). While the emotional health variable is ordinal (almost always/always/most of the time/about half the time/never”) we felt it was conceptually reasonable to treat it as a continuous variable given that the actual distribution of emotional well-being would follow this distribution. We have clarified the latter point in the methods section with the following language:

We built simple logistic regression models to measure the unadjusted odds of association with overcrowded housing and finally constructed a full multivariate regression with all variables of interest to measure the adjusted odds of association. While the emotional health item was collected using a Likert scale, it was modeled as a continuous variable as this was the most conceptually consistent way to consider an individual’s emotional wellbeing.

12. It is not understood how the researchers created the regression model, is it possible to reach the COVID-19 test as a result of living in a crowded house, or is it possible to live in a crowded house because there is no access to the COVID-19 test? Although it is thought that the researchers associated this with low income and therefore it was added to the model, it would be good to state this situation in the text and add it to the discussion with a few words.

Response: We agree that the results are interesting, which is why we are excited to share them with a broader academic audience to spark discussion. In the discussion, we suggest one possible mechanism:

“If a person is living in severely overcrowded housing without the ability or resources to properly quarantine or isolate then the results of a COVID-19 test may not have much impact on behavior, which can diminish incentives for testing.”

13. Lower emotional health is not understood as a predictive factor for overcrowded housing. It seems more logical to us that it occurs as a result of overcrowded housing. Researchers should explain why they add it to the model and how they see it as a predictive factor (Researchers stated in the method section “We conducted a simple logistic regression to examine the predictors for overcrowded housing.”). While it was mentioned in the discussion, "There is evidence that overcrowded housing can have a negative impact on an individual's mental health (27,28). Our findings add to this literature but our narrowed focus on the refugee community highlights that housing conditions can add an extra emotional and mental burden to a population already at high risk of lower emotional/mental health outcomes (29.30).” However, the analysis is a regression analysis of the effect of low emotional health on overcrowded housing.

Thank you for bringing this up. We have updated the title of the table to make it less confusing: “Simple and adjusted odds of living in severely overcrowded housing vs. not living in severely overcrowded housing.”Additionally, we have revised the language in the manuscript and replaced the word predictor with association to prevent confusion.

We have addressed this somewhat in the methods section. Our decision to consider mental health was based on previous research conducted by our team. The decision was also based on consultation with the PANA leadership team, who pointed out that living in substandard (i.e., severely overcrowded) housing can be a source of emotional distress and anxiety for many individuals.

### References

(1) Vandenbroucke, J. P., von Elm, E., Altman, D. G., Gøtzsche, P. C., Mulrow, C. D., Pocock, S. J., Poole, C., Schlesselman, J. J., Egger, M., & STROBE Initiative (2007). Strengthening the Reporting of Observational Studies in Epidemiology (STROBE): explanation and elaboration. PLoS medicine, 4(10), e297. <https://doi.org/10.1371/journal.pmed.0040297>
(2) Cuschieri S. (2019). The STROBE guidelines. Saudi journal of anaesthesia, 13(Suppl 1), S31–S34. <https://doi.org/10.4103/sja.SJA_543_18>
(3) Aneshensel, C. S. (2012). Theory-based data analysis for the social sciences. Sage Publications.
