# Supplementary material for: Overcrowded housing reduces COVID-19 mitigation measures and lowers emotional health among San Diego refugees from September to November of 2020
Source: PLoS One. 2023 Jun 20;18(6):e0286993. doi: 10.1371/journal.pone.0286993 (PMC10281572; doi:10.1371/journal.pone.0286993)
Supplement: Attachment — Submitted filename: Review_PLOSONE_2.10.23.docx [file pone.0286993.s005.docx]

PLOS ONE - Review

February 10, 2023

Title: Overcrowded Housing Reduces COVID-19 Mitigation Measures and Lowers

Emotional Health Among San Diego Refugees from September to November of 2020

This manuscript is on an important topic for refugee health. The analysis is meaningful and would be an important contribution to the literature. Below please find some feedback.

- Abstract
  - The copied and pasted abstract within the submission portal section near key words did not include the same edits as the abstract within the revised manuscript.
  - Rather than saying more than twice the odds and nearly 4 times the odds, use the exact number for the odds. Ex: 3.9 times the odds.
- Introduction
  - I suggest a more specific objectives section that answers who, what, where, and when. The current objectives are more like a study purpose.
- Methods
  - “able to participate in a language” Perhaps change to speaks one of the languages included in the study
  - Incomplete sentence: “Demographic variables such as income from the previous week (Have you worked for money in the previous week), age, year of arrival

to the United States, gender (male or female options only), cohabiting with a partner, number of children (How many children under the age of 18 are you responsible for?), and family size.”

- Was your data normally distributed? This should be checked before running a t test.
- Was it a multivariate regression or multiple regression?
- Results
  - Table 1 figure title – I suggest deleting “Include age, average years in the US, etc
  - I suggest rephrasing: “*were 81% lower odds of reporting living in severely crowded conditions?”*
- Discussion
  - The conclusions are repetitive of findingss and don’t instead focus more on the future
