# Supplementary material for: Overcrowded housing reduces COVID-19 mitigation measures and lowers emotional health among San Diego refugees from September to November of 2020
Source: PLoS One. 2023 Jun 20;18(6):e0286993. doi: 10.1371/journal.pone.0286993 (PMC10281572; doi:10.1371/journal.pone.0286993)
Supplement: Attachment — Submitted filename: Reviewer Comments.docx [file pone.0286993.s006.docx]

PLOS ONE - Review

February 10, 2023

Title: Overcrowded Housing Reduces COVID-19 Mitigation Measures and Lowers

Emotional Health Among San Diego Refugees from September to November of 2020

This manuscript is on an important topic for refugee health. The analysis is meaningful and would be an important contribution to the literature. Below please find some feedback.

·	Abstract

o	The copied and pasted abstract within the submission portal section near key words did not include the same edits as the abstract within the revised manuscript.

*Response: Thank you for catching that, we will update in the author’s portal accordingly.*

o	Rather than saying more than twice the odds and nearly 4 times the odds, use the exact number for the odds. Ex: 3.9 times the odds.

*Response: Based on comments from another reviewer we have restructured our analyses and consequently our results. We have elected to describe the findings more qualitatively in the abstract to ensure that the holistic meaning of the findings is highlighted, rather than the somewhat complex interpretations necessary for standardized path coefficients.*

·	Introduction

o	I suggest a more specific objectives section that answers who, what, where, and when. The current objectives are more like a study purpose.

*Response: We appreciate the reviewer's stylistic suggestion. We have opted to retain the original language to conserve space and because we feel that it is important to highlight the overall purpose of the study in the background before specifying the details the reviewer emphasizes (who, what, where, when) at more length in the methods section.*

·	Methods

o	“able to participate in a language” Perhaps change to speaks one of the languages included in the study

*Response: We have changed this language to: “could speak one of the languages included in the study”*

o	Incomplete sentence: “Demographic variables such as income from the previous week (Have you worked for money in the previous week), age, year of arrival to the United States, gender (male or female options only), cohabiting with a partner, number of children (How many children under the age of 18 are you responsible for?), and family size.”

*Response: We have adjusted this incomplete sentence to: “We also included demographic variables such as income from the previous week (Have you worked for money in the previous week), age, year of arrival to the United States, gender (male or female options only), cohabiting with a partner, number of children (How many children under the age of 18 are you responsible for?), and family size.”*

·	Was your data normally distributed? This should be checked before running ^[1]^a t test.

*Response: Yes, it was normally distributed. Moreover, structural equation model global fit can be evaluated based using tests which do and do not assume normality and so are somewhat robust to these assumptions.*

·	Was it a multivariate regression or multiple regression?

*Response: We have updated our analysis based on comments made by reviewer #4.*

·	Results

o	Table 1 figure title – I suggest deleting “Include age, average years in the US, etc

*Response: We have removed this sentence from the Table 1 title.*

o	I suggest rephrasing: “*were 81% lower odds of reporting living in severely crowded conditions?”*

*Response: We have updated our analysis based on comments made by reviewer 4 that have made this suggestion no longer applicable.*

·	Discussion

o	The conclusions are repetitive of findings and don’t instead focus more on the future

*Response: Thank you. We have removed language that repeats findings and have added more language on recommendations and future directions.*

o	Reviewer #4: Researchers included “access to the COVID-19 test” and “Lower emotional health” as independent factors in the regression model affecting “severely overcrowded housing”, the outcome variable of the article. The researchers created the inverse relationship between dependent and independent factors, and although this inverse relationship was mentioned in the first review, no changes were made in the analysis, only the conclusion sentences were changed. Since the regression analysis was not created appropriately in the article, it is not suitable for publication.

*Response: We concur that originally, it was confusing to follow the relationships between dependent and independent factors. We have since revised the analysis utilizing a structural equation modeling approach which more heavily emphasizes a priori theories of association (see table 3 and figure 1 in revised manuscript). We also accordingly updated our results section and abstract.*

1. Kline, R. B. (1998). Structural equation modeling. *New York: Guilford*.
